# Nouveau design solar septic tank: Reinvented toilet technology for sanitation 4.0

**DOI:** 10.1016/j.eti.2020.100933

**Published:** 2020-08

**Authors:** Thammarat Koottatep, Tatchai Pussayanavin, Chongrak Polprasert

**Affiliations:** a School of Environment, Resources and Development, Asian Institute of Technology, Thailand; b Faculty of Science, Ramkhamhaeng University, Thailand; c Thammasat School of Engineering, Thammasat University, Thailand

**Keywords:** Reinvented toilet technology, WASH, Onsite sanitation, Blackwater treatment, Performance evaluation

## Abstract

The up-flow solar septic tank (UTST) and multi-soil layering (MSL) system has been developed and proposed as “Nouveau Design Solar Septic Tank”. The objective of this study was to verify functionality of the integrated UTST and MSL system for treatment of toilet wastewater (or black water) under actual conditions over a year at the Asian Institute of Technology campus, Pathumthani province, central Thailand. During the operation period which involved fluctuating flow rates, ambient temperatures and black water characteristics, the UTST unit yielded satisfactory performance with the average treatment efficiencies of 92±10% for total chemical oxygen demand (TCOD), 79±10% for soluble chemical oxygen demand (SCOD), 93±9% for total 5-days biochemical oxygen demand (TBOD) and 90±12% for soluble 5-days biochemical oxygen demand (SBOD), respectively, while the MSL unit could remove 95 ±3%, and 88 ±15% of total kjeldahl nitrogen (TKN) and total phosphorus (TP), respectively. The effluent TCOD, TBOD, TKN, nitrite (NO_2_-N), nitrate (NO_3_-N), ammonia (NH_3_) and TP concentrations of the integrated UTST and MSL system were 39±27,8±27,5±5 mg/L, 2±2,39±24,8±9,2±5 and 1±1 mg/L, respectively, meeting the ISO requirements. The removal efficiencies of TCOD, SCOD, TBOD and SBOD exhibited positive correlation with the ratios of TBOD/TKN, TBOD/SBOD and TBOD/TP. With high treatment efficiencies and effluent quality meeting the ISO requirements, the nouveau design solar septic tank has been demonstrated as an innovative technology toward the sanitation 4.0 concept and the Sustainable Development Goal no. 6 (SDG6).

## Highlights

• Reinvented toilet technology is as a sustainable solution for sanitation 4.0.
• To achieve the ISO30500 standard, the nouveau design solar septic tank is proposed.
• Performance of integrated UTST and MLS treatment system in the practical application was evaluated.
• Effluent concentrations and its efficiencies were in compliance with the standards.

## Graphical abstract

**Figure:**
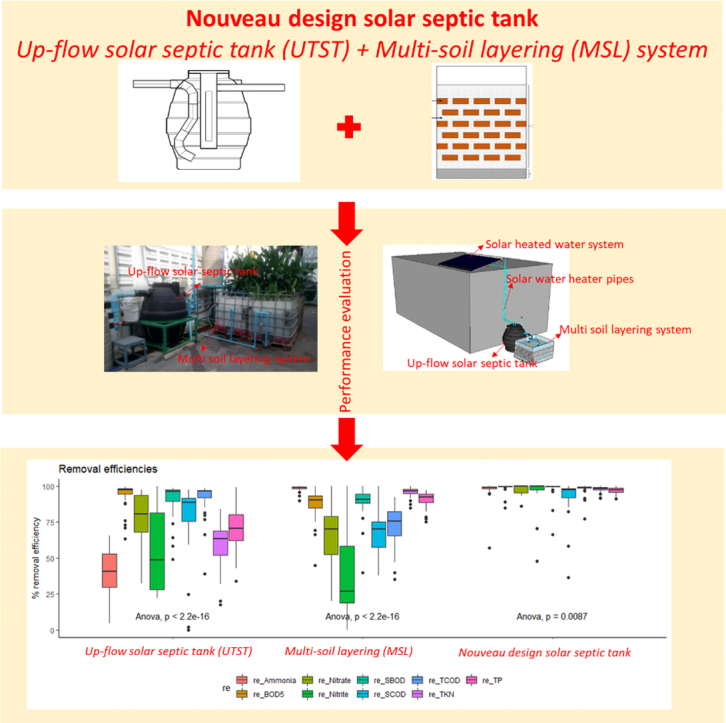

## 1 Introduction

Currently, there are more than 4.2 billion people worldwide who live without access to basic sanitation facilities and safely managed sanitation services, resulting in diarrheal infections and about 1 million child deaths every year (UNICEF and WHO, 2020). Due to poor sanitation, human wastes or waste generating from human activities and containing high level of organic pollutants and pathogens, are discharged into nearby vicinities which can cause environmental pollution and health risks to people. This lack of improper sanitation results in major economic losses through lost productive time, healthcare costs, and mortality (Hashemi and Boudaghpour, 2020). Centralized wastewater treatment as being practiced in most developed countries is one of the solutions to treat those wastes, but, because of its high investment cost and requirement of skilled operation, it seems to be inappropriate for developing countries. In case of Thailand, due to limited budgets, installations of sewer and wastewater treatment plants in several municipalities are still inadequate (Pollution control department, 2020). Alternatively, some cost-effective and implementable onsite solutions such as cesspools and septic tanks are more practically feasible as primary treatment devices to treat sewage or blackwater (Polprasert and Rajput, 1982), but, their effluents still contain relatively high concentrations of organic matters, pathogens and nutrients.

Since the launch of the Act on “Enhancement and Conservation of National Environmental Quality” in 1992, domestic wastewater management in Thailand has relied mainly on the onsite sanitation systems, while some cities are connected to sewerage. Thailand could successfully achieve the Millennium Development Goal (7D) on “providing access to improved sanitation” in early 2004 whereby more than 98% of households are installed with flushing/pour-flush toilets and connected with either septic tank or cesspools/cesspits (MOPH, 2008). There are about 9.9 million m^3^/day of domestic wastewater generated in Thailand, but only 1.05 million m^3^/day are collected and treated at the central wastewater treatment plants, while the remaining 8.85 million m^3^/day are partially treated by the onsite sanitation units. These circumstances occur not only in Thailand but in most developing countries that aim to achieve “Open Defecation Free” whereby human wastes need to be safely managed prior to reuse or disposal. Thus, the remaining challenges toward the Sustainable Development Goal no. 6 (SDG6) on “Safely-Managed Sanitation” could be met through the “Reinvented Toilet Technology” which is in accordance with a concept on Sanitation 4.0 (Koottatep et al., 2019a).

To address the global sanitation problem, the Bill & Melinda Gates Foundation has invested several hundred million US$ to reinvent the sanitation technologies. The Foundation’s goals, since 2011, have focused on development of effective onsite sanitation technologies that poor people can access, and also to create a platform of global sustainability. One of the effective reinvent sanitation technologies is “Solar Septic Tank (SST)” which utilizes solar-heated water to increase temperatures inside the septic tanks and maintain temperature in the range of 35–45 °C. The solar septic tank prototypes have been implemented in several studies to verify their functionality, durability and performance (Pussayanavin et al., 2014, Connelly et al., 2019). To achieve the requirements of the ISO30500 standard for non-sewered sanitation systems, the “Nouveau Design Solar Septic Tank” concept or next-generation treatment systems are proposed. To improve performance of the SST technology in terms of treatment efficiency, cost, and ease of maintenance, the integrated up-flow solar septic tank (UTST) and multi-soil layering system (MSL) system has been developed (technology readiness level 3) and tested in the laboratory (Koottatep et al., 2018, Koottatep et al., 2019b). The objective of the study was to demonstrate and verify functionality of the integrated UTST and MSL system for the treatment of toilet wastewater under actual conditions over a year.

## 2 Material and methods

**Fig. 1:**
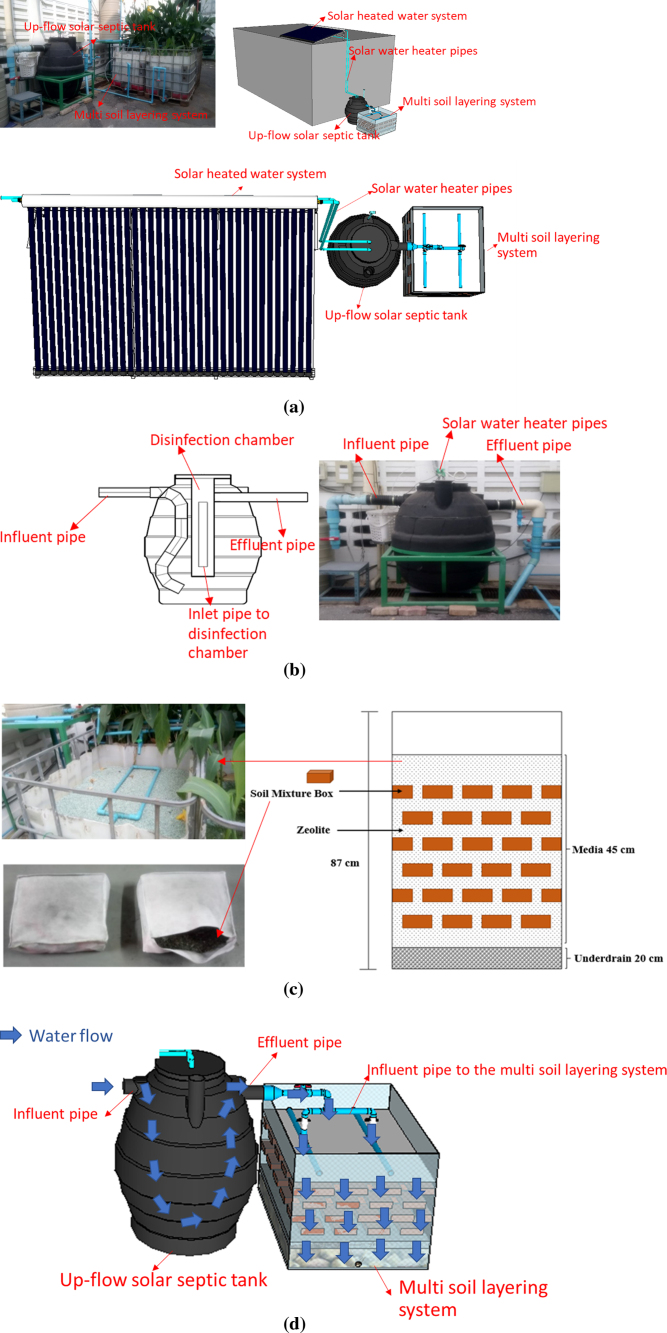
(a) Field testing unit, (b) Upflow solar septic tank unit, (c) Multi soil layer unit and (d) Integrated UTST and MSL system.

A field testing of the integrated UTST and MSL system was conducted under actual conditions of fluctuating flow rates, ambient temperatures and black water characteristics at communal toilets located at the Asian Institute of Technology campus, Pathumthani province, central Thailand. (Fig. 1a). The integrated UTST and MSL unit (Fig. 1) has been in operation since January 2018 with the estimated users of more than 10 people per day per unit. The UTST tank, in a circular shape (Fig. 1b), was made of polyethylene polymer (linear low-density polyethylene material) with an effective volume of 600 L (with dimension of 1000 mm diameter and 1250 mm high), and the designed hydraulic retention time of 24 h and organic loading rate of 4 g/L.day. The up-flow hydraulic mode via a modified influent pipe was designed to improve hydraulic mixing and contact between anaerobic microorganisms and blackwater which should result in better treatment performance than the conventional solar septic tanks. The disinfection chamber, made from polyethylene polymer with dimension of 300 mm diameter and 1000 mm high, was installed in the UTST (Fig. 1b). Temperatures in the UTST were increased by circulating hot water generated from a 12- m^2^ solar water heating device (40-solar vacuum tubes heat pipe, compact pressure model, Shanghai Ruty Energy Co., Ltd, China) through a heat transfer equipment made of spiral copper and recorded hourly using temperature sensors (PT-100 type HDP/7, SWK Technology, Thailand). The generated energy from the solar water heating device was calculated by the following Eq. (1). (Metcalf and Eddy, 2014, Pussayanavin et al., 2020):

(1) $$P\left(kW\right)=\frac{V\left(L\right)\times \gamma \left(kg∕L\right)\times C\left(kJ∕kg\;K\right)\times \Delta T\left(K\right)}{t}$$

Where P = generated energy (kW); V = volume of the UTST (L); γ = density (kg/L); C and specific heat of water (kj/kg °C); ΔT = difference in temperature between time i and j; and t = time (sec).

 The UTST effluent was discharged by gravity without any energy input to the MSL unit designed as a specialized multi-cell subsurface vertical flow which had a dimension of 87 ×94 ×115 cm (working high ×width ×length) (Fig. 1c). The MSL was composed of soil mixture boxes (with a ratio of laterite soil mixed with sawdust and powdered charcoal at 80, 10 and 10%, respectively, on dry weight basis), and the permeable layers were composed of zeolite (clinoptilolite type). Influent and effluent samples of the UTST and MSL units were collected bi-weekly during the one-year operation to monitor the operation and performance of the integrated system. The influent samples were collected by disconnecting the inflow to the UTST via a sampling valve for a period of 24 h where the black water samples were collected in sealed buckets. Various physical–chemical parameters including total chemical oxygen demand (TCOD), soluble chemical oxygen demand (SCOD), total 5-days biochemical oxygen demand (TBOD), soluble 5-days biochemical oxygen demand (SBOD), total kjeldahl nitrogen (TKN), ammonia (NH_3_), nitrite (NO_2_-N), nitrate (NO_3_-N) and total phosphorus (TP) were analyzed according to the Standard Methods (APHA, 2017). The quality of influent blackwater from the communal toilet contained 4139±2721 mg/L of TCOD, 926±556 mg/L of SCOD, 1742±1270 mg/L of BOD, 304±151 mg/L of TKN,169±78 mg/L of NH_3_ and 53±92 mg/L of TP. About 44 samples with two to three replicate for each sample were collected over the times.

**Fig. 2:**
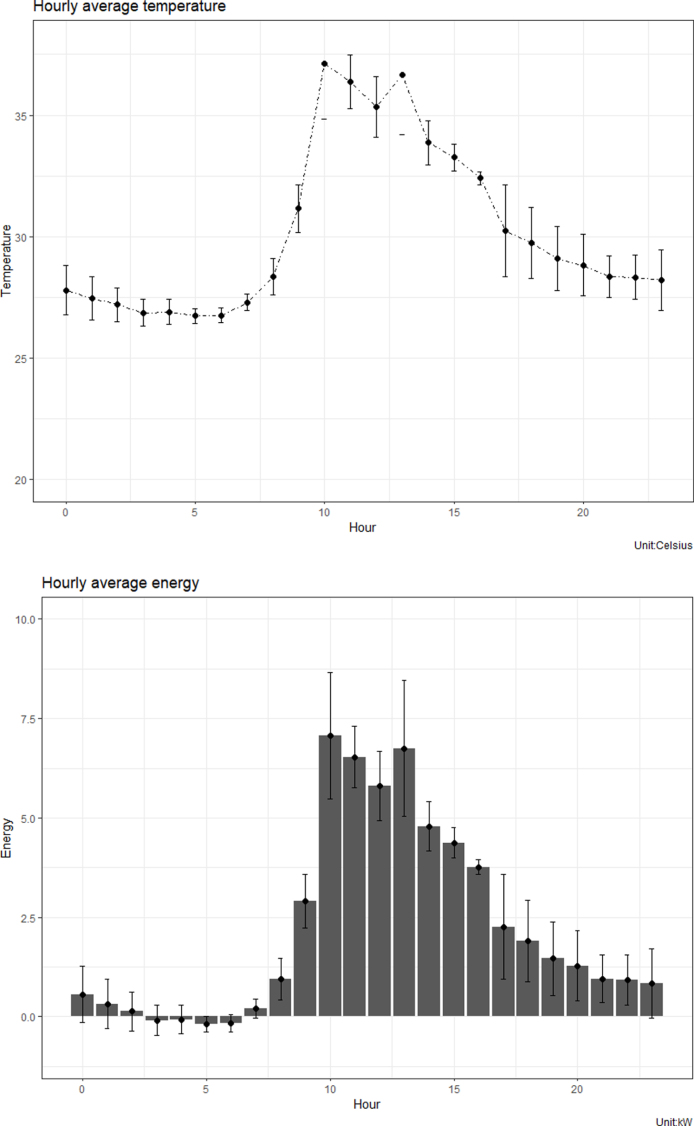
Temperature and energy profile in upflow solar septic tank.

**Fig. 3:**
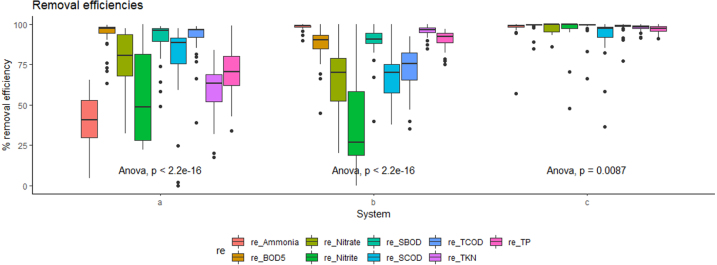
Removal efficiencies of UTST, MSL and integrated UTST and MSL system (a=UTST, b=MSL and c=Integrated UTST and MSL system) (No. of sample: 44) .

**Fig. 4:**
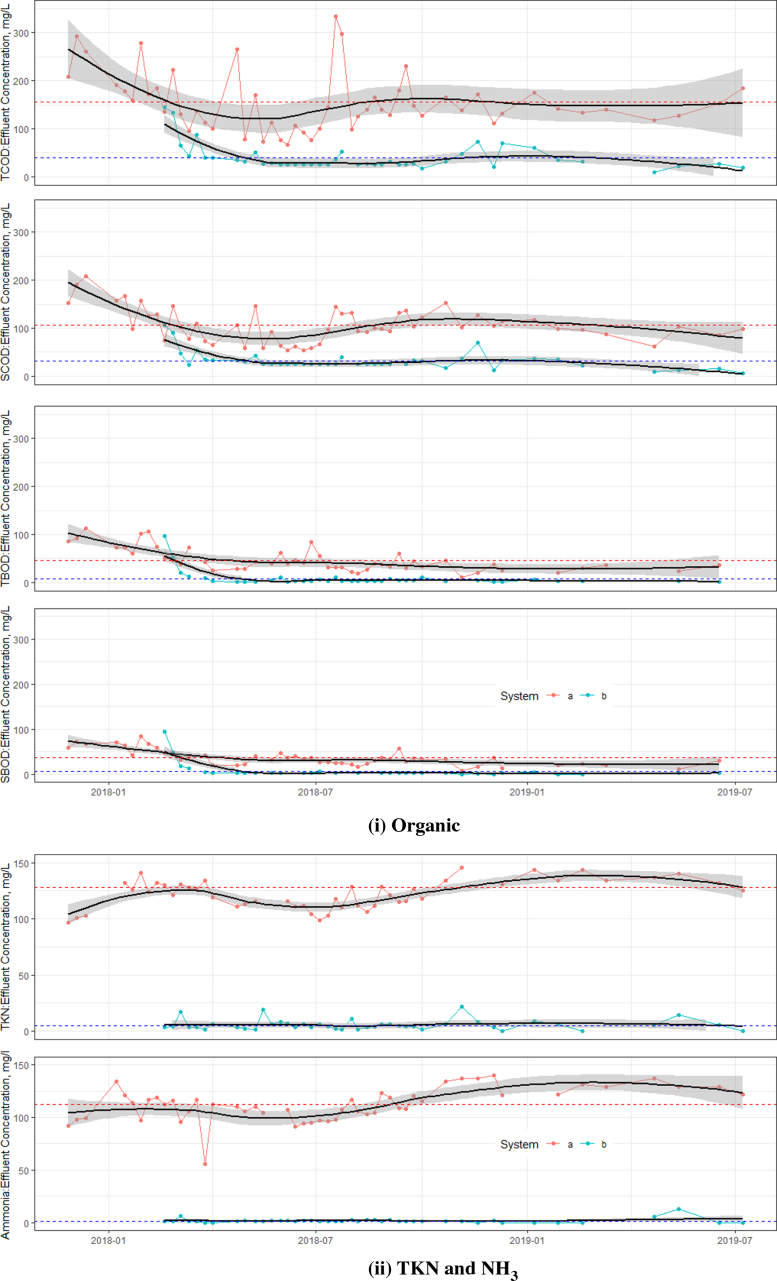

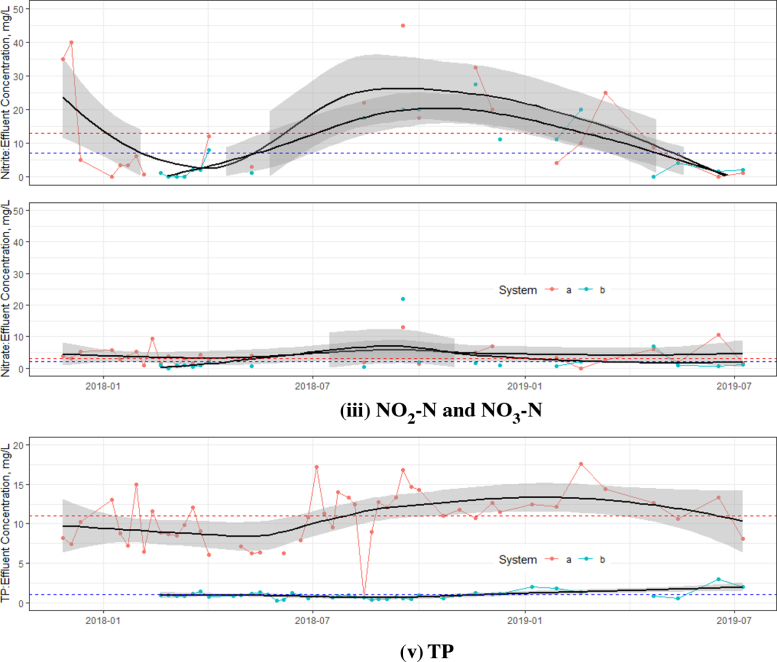
Effluent concentrations from the UTST and MSL units (a = UTST, b = MSL,  = Average concentration (UTST) and  = Average concentration (MSL)). (For interpretation of the references to color in this figure legend, the reader is referred to the web version of this article.)

**Fig. 5:**
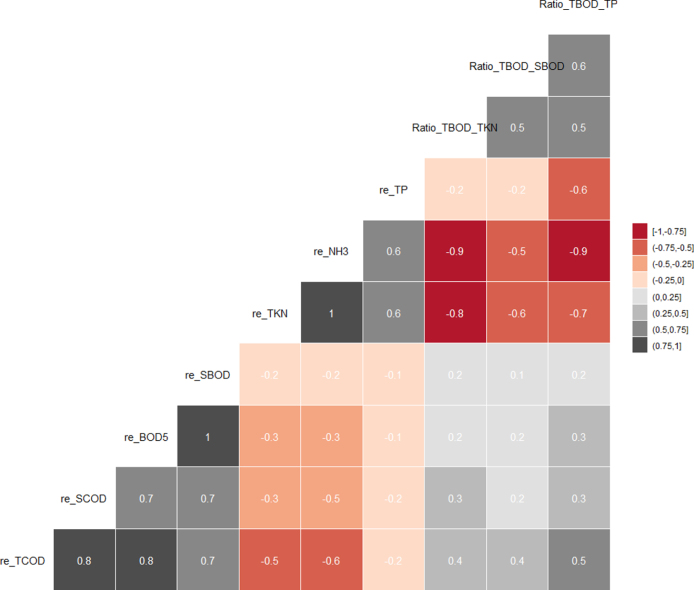
Relationships between removal efficiency and operational factors.

The percent removal efficiency (re) of the UTST unit (a), the MSL unit (b) and integrated​ UTST and MSL system (d) was calculated according to Eqs. (2)–(4).

- Removal efficiency (%) of UTST unit

(2) [(Influent from the communal toilet−Effluent from UTST)∕Influent from the communal toilet]×100

- Removal efficiency (%) of MSL unit

(3) [(Effluent from UTST−Effluent from MSL)∕Effluent from UTST]×100

- Removal efficiency (%) of integrated UTST and MSL system

(4) [(Influent from the communal toilet−Effluent from MSL)∕Influent from the communal toilet]×100

## 3 Results and discussion

During the operation period, the average ambient temperature and solar radiations were about 32 °C and 200 J/m^2^ min, respectively. Temperatures in the UTST equipped with the solar water heating device were in the range of 26–37 °C (Fig. 2). The UTST unit could raise in-tank temperature of about 5–10 °C (on average), compared to the influent wastewater temperature. However, depending on the climatic conditions, the ambient *temperature* and solar *radiation* have daily periodical variations, resulting in the average temperature in the UTST found slightly lower than the thermophilic range (Koottatep et al., 2019b). Depending on ambient temperatures, climatic conditions and operation periods, the energy inputs to the UTST were found in the range of 1–7 kW, with the average being 4 kW. The negative values of more than 2 kW were due primarily to energy losses from the UTST caused by effluent flow and heat dissipation from the system and during the monsoon period.

Fig. 3 shows that the treatment performance of the integrated UTST and MSL unit during the one year operation period with the average treatment efficiencies of 92±10% for TCOD, 79±10% for SCOD, 93±9% for TBOD and 90±12% for SBOD, respectively. Probably due to hydraulic mixing and relatively high temperatures in the UTST, there were high removal of COD and BOD in the UTST. According to Vrieze and Verstraete (2013), the pulse feeding mode of organic matter can encourage higher functional stability in biological digestion. Therefore, the UTST reactor under intermittent feeding or flushing mode, which represents actual people’s behavior in toilet using, should be effective in treating organic matter in the blackwater. Koottatep et al. (2019b) and Farajzadehha et al. (2012) reported the upflow anaerobic operation in wastewater treatment could lead to more contact between wastewater and sludge that resulted in better decomposition of organic matter. The removal efficiencies of TKN and TP in the UTST were 59±15% and 70 ±15%, respectively, lower than those of the organic matters. Due to the anaerobic condition prevailing in the UTST reactor, the TKN and NH_3_ removal efficiencies for all the HRTs were relatively low, suggesting requirement of the MSL units for further TKN and NH_3_ removal from the UTST effluent samples. However, there were 95 ±3%, and 88 ±15% removal of TKN and TP, respectively, by the MSL unit. The results of Fig. 3 also show the superior performance of the MSL unit in removing NH_3_ (98 ±2%) when comparing with UTST unit (39±16%). It could be hypothesized that there were more abundance of ammonia oxidizing archaea (AOA) and ammonia oxidizing bacteria (AOB) in the MSL beds which presumably oxidized NH_3_ to become NO_3_-N which would be further denitrified to become nitrogen gas by the denitrifying bacteria. To better understand about the medias and water relationship in the MSL unit, mass balance analysis is recommended for the further study. The average removal efficiencies of TCOD, SCOD, TBOD and SCOD of the integrated UTST and MSL units during the operation period were found in the range of 90%–99%, and there was a significant difference (p>0.05) among the data of each parameter. By integrating between the UTST and MSL, the average nitrogen and phosphorus removal efficiencies satisfy with the requirements of the ISO 30500 standard.

The effluent characteristics from the UTST were still in the same magnitude of 115±61 mg/L of TCOD, 107±35 mg/L of SCOD, 46±24 mg/L of TBOD and 36±17 mg/L of SBOD, meeting the TBOD discharge standards of Thailand (MNRE 2010), as shows in Fig. 4. Although Polprasert et al. (2018) and Pussayanavin et al. (2014) reported that the solar septic tank could remove TCOD and TBOD better than the conventional solar septic tank, the UTST with up-flow feeding mode was found to have better treatment performance (Fig. 4). An increase in removal rates by MSL unit could produce the treated effluent in compliance with the ISO requirements. The effluent TCOD, TBOD, TKN, NO_2_-N, NO_3_, NH_3_ and TP concentrations of the MSL were 39±27, 8±27, 5±5mg/L, 2±2, 39±24, 8±9, 2±5 and 1±1 mg/L, respectively. These results indicated that the nouveau design solar septic tank could effectively promote the biodegradation of the organic matters, and convert those nutrients into final products, resulting in high treatment performance. During the 1-year operation period, the average effluent TCOD concentration was lower than 50 mg/L, which was about 39±27 mg/L and meeting the ISO 30500 Category A standard. Compared to TN and TP requirements by the ISO30500 Category A i.e. 70% and 80% removal efficiencies of TN and TP, respectively, the nouveau design solar septic tank can satisfactorily achieve an overall TN (the sum of TKN, NO_2_-N and NO_3_-N) and TP removal efficiencies of 95% and 98%, respectively. A study by Koottatep et al., 2014a, Koottatep et al., 2014b on the same solar septic tank operating at the temperatures of 35–40 °C found the reduction of Escherichia coli (*E. coli*) densities to be about 1–2 log at 40 °C. Further studies on the pathogen inactivation efficiency of the UTST and MSL units are strongly recommended to validate the technical feasibility and its performance. For the prototype of a single family, the UTST tank and MSL units are estimated to cost about US$ 300–1000, while the commercial solar water heating device to heat the UTST would cost about US$ 2000, or the total investment of the integrated system would be in the range of US$ 2000–3000. However, it should be noted that the field test (or prototype unit) should thus be an integral part of the design process and not be used only to consider as a final product and the commercialized product.

Many operational factors have important effects on removal efficiencies in the nouveau design solar septic tank, as shown by the relationship in Fig. 5. The removal efficiencies of TCOD, SCOD, TBOD and SBOD exhibited positive correlation with the ratios of TBOD/TKN, TBOD/SBOD and TBOD/TP. The ratio between TBOD/TP was found to correlate most with the removal efficiencies of TCOD (R${}^{2}=0.5$, P < 0.001), TKN (R^2^ = −0.7, P < 0.001), NH_3_ (R^2^ = −0.9, P < 0.001) and TP (R^2^ = −0.6, P < 0.001). During the operation period, the main reasons for the low TKN and NH${}_{3}$ removal in these UTST and MSL units could be the low TBOD/TKN ratios of the influent black water. Increasing the TBOD/TKN ratios of the influent feed could be achieved through installing urine-diversion toilets to separate the urine (containing high NH_3_-N) from the influent black water.

## 4 Conclusions

Having high treatment efficiencies and effluent quality as reported above, the nouveau design solar septic tank has been demonstrated and verified to be an innovative technology to fulfill the sanitation 4.0 and should be considered as a new sanitation paradigm for global level to improve the region’s environment and achieve the SDG6. Based on the results of this study, specific conclusions can be made as follows:

1. The UTST unit yielded satisfactory performance with the average treatment efficiencies of 92±10% for TCOD, 79±10% for SCOD, 93±9% for TBOD and 90±12% for SBOD, respectively.
2. The performance of the MSL for TKN and TP removals was 95 ±3%, and 88 ±15, respectively, better than those of the UTST.
3. Further removal for ISO requirement was achieved by treating the effluent of the UTST through the MSL. The effluent TCOD, TBOD, TKN, NO_2_-N, NO_3_-N, NH_3_ and TP concentrations of the MSL were 39±27, 8±27, 5±5 mg/L, 2±2, 39±24, 8±9, 2±5 and 1±1 mg/L, respectively.

## CRediT authorship contribution statement

**Thammarat Koottatep:** Conceptualization, Funding acquisition, Investigation, Supervision, Resources, Writing - review & editing. **Tatchai Pussayanavin:** Conceptualization, Data curation, Formal analysis, Investigation, Methodology, Software, Visualization, Project administration, Writing - original draft. **Chongrak Polprasert:** Conceptualization, Supervision, Investigation, Validation, Writing - review & editing.

## Declaration of Competing Interest

The authors declare that they have no known competing financial interests or personal relationships that could have appeared to influence the work reported in this paper.
